# Round the Hospitals

**Published:** 1919-05-03

**Authors:** 


### ROUND THE HOSPITALS.

There is at present no association which is representative of the heads of the nursing profession, and we welcome the activity which is being exhibited in the direction of meeting this serious need by the action on the part of the matrons of the leading London and provincial training-schools. Already a resolution has been passed asserting the importance that such an association shall be formed of trained nurses who hold, or have held, the position of matron or superintendent of hospitals or institutions concerned in the training of nurses and the care of the sick. A Provisional Committee has been formed for the purpose of preparing a draft constitution to be laid before a general meeting of those matrons interested in the scheme, which will be held at St. Thomas's Hospital—by kind permission of the treasurer and governors—on May 8, at 3 p.m., in order to establish such an association. Miss Lloyd Still (St. Thomas's Hospital) and Miss Cox-Davies (Royal Free Hospital) are the honorary secretaries, to whom a notification of being present should be addressed. This step has not been taken a moment too soon, and every matron who has the welfare of her profession at heart—and who has not?—should make it her business to be present at the meeting on 8th inst. at St. Thomas's Hospital and support her sisters, who have exhibited great public spirit and are entitled to leadership. We hope the matrons of the large provincial hospitals will be requested by their committees to attend this meeting and take part in its proceedings, for the more widely the great training-schools are represented the better.

If hospital committees wanted any stimulus to arouse themselves and move Parliament to awaken to the dangers which threaten the future of nursing in the United Kingdom, unless they use their influence to prevent the injustice to trained nurses which is being attempted by the relatively small section who have set themselves to sow disunion throughout the nursing world for their own purposes, it will surely be their patriotic duty to oppose and help defeat the Central Committee's Registration Bill now before the House of Commons as it stands, for little but evil must otherwise result. It is essential to sound nursing progress for the great hospitals and training-schools, and for every trained nurse who values her independence and the best interests of her profession, to work with a will to bring the true facts of the present position to the notice of every member of the Legislature without further hesitation or delay. The people of these islands are wholeheartedly and unanimously grateful to British nurses for the splendid work they have done during the war. Is there a member of either House of Parliament who will not willingly devote time to an examination of the mischief and injustice with which the nursing profession is threatened at the moment through the few irreconcilable spirits who have set themselves to mislead Parliament and capture the control of British nurses, their education, training, and discipline? We have confidence that Parliament, now that its attention has been called to the matter, will unite in defence of our nursing sisters, and secure the passing of a Registration Bill free from the evils with which they are threatened by the Central Committee's Bill, which represents the interests of the disunionists, and tyranny for British nurses.

The Army Council has conveyed to all members of Voluntary Aid Detachments who have worked so hard and with such devotion their appreciation of the valuable help so unstintingly given. They warmly commend the keenness, self-sacrifice, and devotion to duty displayed at all times and in all circumstances and have put it on record that without the help of these workers the difficulties regarding the furnishing of adequate care and attention to the sick and wounded would have been immensely increased. We think it would be nearer the truth to say that without this voluntary aid the difficulties would have proved insurmountable. In conclusion the Army Council express their "deep sense of gratitude to these devoted helpers in the great cause of humanity." An illustration of devotion to duty "at all times and in all circumstances" was afforded by Miss Alice Batt, V.A.D., to whom the King presented the Albert Medal on April 25 for gallantry in saving life. She was in attendance on the surgeon who was performing an abdominal operation on the occasion when fire broke out at No. 36 Casualty Clearing Station at Rousbrugge in October. The lights went out and the room became filled with smoke, but the operation was continued with an electric torch, and Miss Batt went on handing instruments and threading needles with steadfast calmness till the surgeon had finished his task, when she assisted to carry men from the burning wards to a place of safety.

The Rev. A. Lombardini will give a course of three addresses this week, May 1, 2, and 3, at special services to be held for nurses and for those interested in the nursing profession at the Church of St. Martin-in-the-Fields, Trafalgar Square, at 3.30 p.m. The subject of the addresses is "Divine Legacies." There will be a collection each day in aid of the Nation's Tribute Fund for Nurses. The Nurses' Week would not have been complete without the inclusion of the devotional aspect and the opportunity afforded will be heartily welcomed by both nurses and their friends.

A scottish Sub-Committee has been formed by the Nurses' Resettlement and Demobilisation Committee appointed by the Minister of Labour. The work which this Resettlement Committee has to perform is exceedingly intricate, dealing as it does with the future prospects and present circumstances of thousands of nurses. Any failures are loudly heralded in the lay press. The successes resulting from patient investigation and attention to detail commonly pass unnoticed. The new sub-committee is charged with controlling the list of Scottish nurses

**ROUND THE HOSPITALS—(*continued*).**

desiring work in Scotland, and its office is at the Employment Department, Ministry of Labour, 112 George Street, Edinburgh, where inquiries should be addressed. Nurses who want to take any form of supplementary training will receive advice and assistance. Among the members of the sub-committee are Miss Merchant, Matron of the Eastern District Hospital, Glasgow, representing the Local Government Board; Miss Melrose, R.R.C., Royal Infirmary, Glasgow (Scottish Matrons' Association); Miss White (Q.V.J.N.I.); Miss Gill, R.R.C., Royal Infirmary Edinburgh (College of Nursing); Miss M. M. Paterson (National Health Insurance); Miss Younger, O.B.E. (Ministry of Labour). Nursing V.A.D. members and special military probationers can be put in the way of obtaining further work or training by making application on Form Z27A (Nurses), which can be obtained from their senior officers, to the Nurses' Demobilisation Committee, 16 Curzon Street, Mayfair, W. 1. They can be interviewed either in London or in Scotland.

---

THE Committee of the Royal Portsmouth Hospital has approved a scheme drafted by the matron, Miss A. E. Alcock, R.R.C., whereby members of the nursing staff have one day off duty in seven and also increased off-duty time each day (making 56 working hours weekly for staff nurses and probationers and 50 hours weekly for sisters), as soon as the necessary additional nurses have been engaged. Probationers' salaries have also been raised to £16, £20, and £24; staff nurses' to £40; night sisters', £55 to £60; ward, theatre, and casualty sisters', £50 to £60; assistant matrons', £70; *x*-ray and massage sisters', £60.

---

THE eight-hour day is to be inaugurated in Ireland in the Royal Victoria Hospital, Belfast. This announcement was made at the meeting of the board of management held last month, with Professor Lindsay, M.D., in the chair. Very considerable expenditure is involved in the alteration, which cannot take place until extra nurses have been engaged and the number of domestics increased.

---

THE Committee of the Gravesend Hospital have raised the matron's salary from £100 to £125. Sisters, whose maximum salary is now £45, will commence at this figure and rise to £55. Probationers are to receive £12, £16, £20. Before the war the sister's salary was £30. How much of the present rise in salaries is to be attributed to war prices is still hard to ascertain.

---

THE latest institution to join the "One Day in Seven" movement is the Essex County Hospital at Colchester, where the working week will be reduced to six days as soon as the number of probationers can be increased sufficiently. Salaries for nurses in training have been raised to £15, £20, £25.

THE *Trained Nurse* of New York has a serious indictment of hospital managers in the April number, under the heading of "Some Factors which cause Nurses' Strikes." In one case which came to the writer's notice a strike was avoided at the last hour by granting the nurses' demands, which comprised better food, release from ward-scrubbing and dish-washing, two blankets instead of one for their beds, and a reception room for their friends. We do not know of any British hospital where nurses are required to do the scrubbing or pantry work, nor do we believe this to be common in the United States.

---

THE Derbyshire County Nursing Association has long been conducted on good lines, and at the twenty-fifth annual meeting excellent progress was still the note. There are now eighty nurses working for the Association, seven new nursing societies, embracing sixteen villages, have been added during the year, and the work of training candidates continues. The expenditure amounts to £1,000 a year, and a successful "Need for Nurses' Day" held last year, induced the Committee to believe this could be made an annual feature and bring in a steady income. A scheme for giving bonuses to the nurses after every five years of work is under consideration. There are still many places in Derbyshire unprovided with district nurses, so there must be no slackening of effort.

---

THE authorities of St. Thomas's Hospital are extending co-operation to a scheme, formed under the Poona Seva Sadan, for giving post-graduate courses in midwifery and other subjects to selected female candidates who have qualified as midwives, or sub-assistant surgeons at Poona. Great efforts are being made to improve the conditions of nursing by Indian women, and a hostel is being provided for them at Calcutta under the auspices of a strong British and Indian Committee. There are already three Calcutta hospitals where native women can be trained, two of which offer a three-year course. The main difficulty after being trained is to find suitable quarters, and these the hostel will supply. A guild for Indian nurses was formed two years ago under the Y.W.C.A., and this organisation will undertake the management; nurses will have free board and receive a fixed salary as in an English nurses' institute. The need for a strong nursing reserve for use in epidemics, war, or times of public danger has been overwhelmingly manifested in every part of India. But the normal requirements are as yet very far from met by the nurses available, and lack of adequate facilities for training suitable candidates continues to be the great drawback to the development of Indian nursing.

---

THE Fourth Southern General Hospital at Plymouth celebrated its demobilisation on April 23 by a farewell At Home given in the Assembly Rooms of the Royal Hotel. The hostesses were the Principal Matron, Miss Small, R.R.C. and the Matrons, Miss C. A. Tait McKay, R.M.C., and

**ROUND THE HOSPITALS**—(*continued*).

Miss Priestman, R.R.C., together with the nursing staff of the hospitals which made up the 4th Southern General. The occasion was a memorable one. For four and a-half years Plymouth has been the centre of finely organised medical and nursing work for the wounded and sick, conducted by the Territorial Force Nursing Service under Colonel Webber and (later) Lieut.-Colonel J. Elliott Square. The people of Plymouth have aided generously so far as their services could be utilised, and their regret at the closing down of the hospital and the departure of the nursing staff has been manifested in many graceful ways. The efficiency which has marked the entire hospital administration from first to last and in particular the able and harmonious manner in which the three matrons organised their respective nursing departments was the universal theme, and has elicited unstinted admiration in the town and the local press. The guests, numbering some 250, included numerous friends of the hospital, the officers of the garrison and port, and prominent people in the town.

It is the Anglo-Belgian Union which has charge of the arrangements for the re-burial of Miss Edith Cavell. The Committee has been in communication with the Vice-Dean and Chapter of Norwich Cathedral, and after careful consideration a site has been agreed upon for her tomb outside the walls of the cathedral but in close proximity to it, in an enclosed but unoccupied space known as Life's Greens, about ten yards to the eastward of St. Luke's Chapel. Nurses should not fail to procure the *Daily Telegraph* of April 21 and 22, in which Mr. Brand Whitlock, the American Minister in Belgium, gives a detailed description of Nurse Cavell's work in Belgium, the heroic deeds she performed in rescuing young Belgian, French, and English soldiers from the Germans, her trial, condemnation, and death. We trust the story will shortly be reprinted, for it should be in every hospital library. Many new facts transpire in this record, and it is by far the most valuable document available of the full life of Miss Cavell There will be a military procession from Victoria Station to the Abbey on the arrival of the funeral cortège from Dover, and again after the Abbey service to Liverpool Street, on May 15, whence the body will be taken by train to Norwich, and thence direct to the cathedral.

A complaint has made itself heard that nurses' annuities, if over £130, are taxed at the rate of 6s. in the £, and that "you are told you can get part back, but never anything like the whole amount that should be returned." Several instances of this sort are quoted, in which "the nurse past fifty is so taxed that it is impossible for her to live on her income." The truth is that the possessor of an income of, say, £160 a year is liable merely for the tax on £40, £120 being allowed free of income tax. On this £40 she is expected to pay 3s. in the £, or £6 in all. The process of getting back the excess tax is very simple, and any business man would explain it. Needless to say, the authorities may be relied on to refund to the uttermost farthing. It is, however, a real hardship that the income tax should be deducted in advance at the 6s. rate, and several large insurance offices have now succeeded in obtaining a concession from the Government that, on receipt of a signed declaration from the holder of a policy that her total income does not exceed £400, income tax will only be deducted at the 3s. rate. By special arrangement with the authorities, income tax is not deducted from annuities paid to policy-holders of the Royal National Fund for Nurses resident in the United Kingdom.

The Edith Cavell Fund in Melbourne, which resembles in its object the Nation's Tribute Fund, has now reached the sum of £9,000, and the interest is available for cases of sickness and distress among the nurses of the Dominion. Adequately to deal with all cases it should not be less than £50,000, and a strong appeal for additional subscriptions is now being made. The number of nurses who left Australia for service in the war totalled 3,000, of whom one-third were Victorians.

A well-attended meeting of the Bedfordshire Hospital Guild took place on March 27, with Dr. Gifford Nash in the chair. The Guild was started eleven years ago to aid the hospital, and this year 776 articles have been contributed by the Needlework Guild, £406 has been raised by house-to-house collections, and £52 contributed by places of amusement. In all, over £11,000 has been raised by the Guild, or almost double the amount collected in 1913. These figures are deeply significant.

No fewer than 140 nurses from the Northern Hospital, Winchmore Hill, were granted the franchise on their application at Southgate Revision Court. Of this number seventy-three were under thirty and therefore had to be content with the Local Government vote only. The nurses were inspired to this public-spirited action by a new-comer who, having gained the franchise herself, awoke interest among the others. The incident is valuable. It shows what can be done by the influence of one nurse.

The dangers of match-boarded iron buildings were illustrated on April 25, when the Windsor Isolation Hospital was destroyed by a fire which broke out in the nurses' quarters through the over-heating of a stove. There were three patients in the building who were safely removed, and there was no loss of life. But all the bedding, clothing, and personal property of the nurses was destroyed, for the building was gutted within an hour of the fire being discovered. It is not creditable to the Windsor Rural District Council that this purely temporary structure should have been left standing for twenty-seven years.

